# Predicting Gene Ontology Function of Human MicroRNAs by Integrating Multiple Networks

**DOI:** 10.3389/fgene.2019.00003

**Published:** 2019-01-29

**Authors:** Lei Deng, Jiacheng Wang, Jingpu Zhang

**Affiliations:** ^1^School of Software, Central South University, Changsha, China; ^2^School of Computer and Data Science, Henan University of Urban Construction, Pingdingshan, China

**Keywords:** miRNA function annotation, miRNA co-expression, global heterogeneous network, latent representations, multi-classification

## Abstract

MicroRNAs (miRNAs) have been demonstrated to play significant biological roles in many human biological processes. Inferring the functions of miRNAs is an important strategy for understanding disease pathogenesis at the molecular level. In this paper, we propose an integrated model, PmiRGO, to infer the gene ontology (GO) functions of miRNAs by integrating multiple data sources, including the expression profiles of miRNAs, miRNA-target interactions, and protein-protein interactions (PPI). PmiRGO starts by building a global network consisting of three networks. Then, it employs DeepWalk to learn latent representations as network features of the global heterogeneous network. Finally, the SVM-based models are applied to label the GO terms of miRNAs. The experimental results show that PmiRGO has a significantly better performance than existing state-of-the-art methods in terms of *F*_*max*_. A case study further demonstrates the feasibility of PmiRGO to annotate the potential functions of miRNAs.

## Introduction

MicroRNAs (miRNAs) are endogenously small non-coding RNAs of about 21–25 nucleotides and play important roles in gene regulation, via base-pairing mRNA molecules with complementary sequences for cleavage or translational repression (Bartel, 2004; Huang et al., 2011; Yao et al., 2018). Some of the biological processes within which miRNAs are involved include development, differentiation, apoptosis, and viral infection (Miska, 2005). In addition to their importance in biological processes, miRNAs are also valuable biomarker candidates for specific diseases, including Alzheimer's disease (AD) (Esteller, 2011). Currently, the identification of unknown miRNA functions is an essential goal of miRNA research. Research on miRNA function focuses on the experimental determination field. miRNA function is primarily identified by the up-regulation or down-regulation of miRNA expression and its target genes (Zhu and Helliwell, 2010). However, experimental methods for the identification of miRNA functions are considerably expensive and time-consuming.

Recently, computational methods have been proposed to solve those difficulties. These methods elucidate miRNA functions by analyzing the functions of target genes or promoters, which are determined by miRNA-related expression (Pandey and Krishnamachari, 2006; Wei et al., 2012). These methods include TargetScan (Agarwal et al., 2015), Miranda (Enright et al., 2003), PITA (Kertesz et al., 2007), and DIANA-microT (Maragkakis et al., 2009). Many of the tools used are based on the sequence alignment of the miRNA seed region, which allows for the determination of the putative binding sites (Maragkakis et al., 2009). However, the prediction results of these tools are unsatisfactory for two reasons: first, the majority of the prediction data of the miRNA target are negative, and the predicted data are not sufficient enough; second, these tools only concentrate on sequence information (Ulitsky et al., 2010) and ignore other useful information, such as miRNA expression data. Therefore, the results are easily affected by negative samples leading to poor results. In a time of increasing high-throughput sequencing, a massive amount of miRNA-seq data is accumulating, however, the analysis of this data remains a significant challenge. miRNA expression determines function, which is also crucial for discovering molecular mechanisms of human gene regulation (Panwar et al., 2017). Backes et al. (2016) developed a novel miRNA annotation tool which provides rich functionality in terms of miRNA categories based on miRNA enrichment analysis. However, miEAA does not take the importance of miRNA co-expression into account. Generally, multiple miRNAs might jointly regulate a target gene, and a miRNA may regulate hundreds of different target genes (Krek et al., 2005; Friedman et al., 2009). The potential associations between miRNAs are also vital to understand the miRNA functional mechanism and to annotate functions of miRNAs. Moreover, miEAA ignores the interactions between miRNA and target gene production (e.g., protein), which provides useful information for predicting the functionalities of miRNAs.

In this paper, we take full advantage of miRNA expression profiles, miRNA-target gene interactions, which are experimentally validated, and protein-protein interactions data. Moreover, a global miRNA-protein network is constructed by integrating these three data sources. Secondly, we employ DeepWalk (Perozzi et al., 2014), an approach used for learning potential representations of nodes in a network, to extract the network features of the global heterogeneous network. Based on these features of the global network, we build an SVM-based classifier for each miRNA to annotate their GO functions. The proteins with Gene Ontology annotations in the GOA database (Huntley et al., 2009) are utilized to train SVM classifiers. Finally, we evaluate our method by applying it to an independent dataset. The results show that our method, PmiRGO, achieves a maximum F-measure of 0.310 and outperforms the other state-of-the-art method, miEAA (Backes et al., 2016).

## Materials and Methods

**Figure 1:**
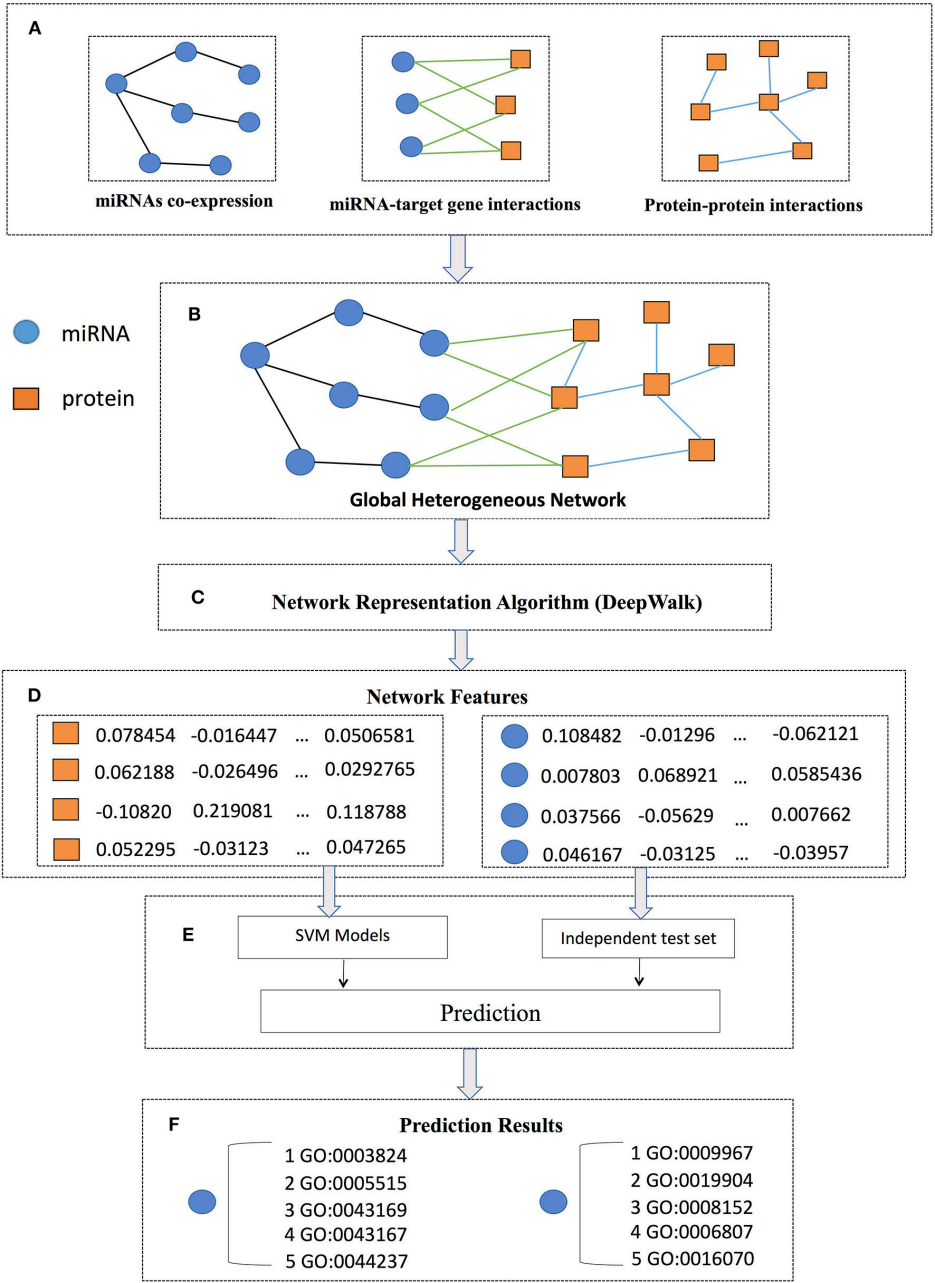
PmiRGO flowchart. It consists of six steps: **(A)** three networks (miRNA co-expression network, miRNA-target interaction network, and PPI network) were constructed according to the co-expression profiles, miRNA-target gene interactions, and protein-protein interactions, respectively. **(B)** By mapping the target genes into PPI network, the three networks were integrated to build a global heterogeneous network. **(C)** DeepWalk was employed to learn the latent representations of the network as features of the global heterogeneous network. **(D)** For each miRNA or protein, a feature vector was obtained. **(E)** SVM models were trained and the miRNA2GO-337 dataset were used to evaluate the performance. **(F)** The GO annotations of each miRNA in the miRNA2GO-337 dataset were predicted.

The flowchart of PmiRGO is illustrated in Figure 1. As shown in step A, we first downloaded the miRNA co-expression profiles, miRNA-target interactions, and protein-protein interactions (PPIs) to construct the miRNA co-expression network, miRNA-target interaction network, and PPI network, respectively. Then, the three networks were integrated to build a global heterogeneous network by mapping the target genes into PPI network in step B. We employed DeepWalk to learn the potential representations of the networks as the features of the global heterogeneous network in step C. In step D, we mapped the IDs of miRNAs and proteins to the corresponding nodes in the features. After that, we trained SVM models for each miRNA and used the miRNA2GO-337 dataset to evaluate the performance of the multi-classification models in step E. In the final step F, the GO annotations of miRNAs in the miRNA2GO-337 dataset were predicted.

### Materials

In this study, we downloaded the miRNA expression data, PPI data, and miRNA-target interactions from different databases, from which a total of 2,588 miRNAs and 18,143 proteins were retrieved. The details are as follows.

#### miRNA Expression

The miRNA expression data were downloaded from the miRmine database, containing expression profiles collected from several publicly available miRNA-seq datasets, as well as detailed information regarding different miRNAs (Panwar et al., 2017). This database consists of expression profiles of 2,822 precursor miRNAs, each containing a total of 135 columns of expression values from different human tissues. Note that a mature miRNA may have two or more precursor miRNAs, in our work; the expression profiles of one mature miRNA derived from different precursor miRNAs were averaged as the expression values of this mature miRNA. As a result, 2,588 miRNA expression profiles were obtained. We then calculated the Pearson's Correlation Coefficient (PCC) scores as the co-expression similarity of the expression profiles between each pair of miRNAs (Zhang J. et al., 2017). We constructed a miRNA co-expression network according to the co-expression similarity values. As the PCC scores were used as the weight of the edges in the network, the negative PCC values were removed.

#### Protein-Protein Interactions

The PPIs were obtained from the STRING database V10.0 (Szklarczyk et al., 2014). These interactions were collected from not only biological experiments but also text mining and computational prediction approaches. The overall scores of these interactions were obtained from single or multiple clues with high probability. The number of PPI entries retrieved from 18,143 proteins was 7,866,428, which were then used to construct a PPI network. Each entry of the PPI network consists of protein A, protein B, and corresponding predicted score. The higher the predicted score of an entry, the higher the probability that two proteins in the entry are considered to interact. In our work, we treat the predicted score as weight of the edge between two protein nodes in the entry.

#### miRNA-Target Interactions

We retrieved miRNA-target interactions from the miRTarBase database of release 7.0 (Hsu et al., 2010). The database provides a gold standard resource of experimentally validated microRNA-target interactions, which were manually collected. We extracted 355,684 different high quality experimentally validated miRNA-target interactions among 2,588 miRNAs and 18,143 target genes to build the miRNA-target interaction network after removing the duplicate and out-of-range entries.

### Methods

#### Constructing the Global Network

Three heterogeneous networks, including the miRNAs co-expression network, the miRNA-target interaction network, and the PPI network, were built as described above. The construction of the miRNA co-expression network is based on the hypothesis that miRNAs with similar expression patterns also share similar functions or biological pathways (He and Hannon, 2004; Zhang Z. et al., 2017). The PCC scores were computed to represent the similarity between two miRNAs and the values represent the weights of the edges in the miRNA co-expression network. Moreover, growing evidences have revealed that miRNAs have identical or related functions to their interacting target genes with a significant probability (Bartel, 2009). Hence, the three component networks were integrated to infer the functions of miRNAs. Assuming that *M, P*, and *MP* denote the adjacency matrices of the miRNA co-expression network, PPI network, and miRNA-target interaction network, respectively, the global network can be formulated as:

(1) $$\begin{array}{r} G=\left[\begin{array}{cc} M & MP\\ MP^{T} & P \end{array}\right] \end{array}$$

Here, T in *MP*^*T*^ represents the transpose.

#### Learning Latent Representations of Nodes

In order to obtain the low-dimensional topological information of the vertices of the global heterogeneous network we constructed above, DeepWalk was used to learn the potential representations of miRNAs and proteins in networks (Perozzi et al., 2014). This unsupervised method based on graph learns features that define the graph structure independently of the distribution of the labels (Bengio et al., 2013). DeepWalk uses information extracted locally from truncated random walks for the learning of potential representations by regarding walks as sentences.

We treated the global heterogeneous network as an undirected graph *G* = (*V* , *E*) that *V* denotes the set of biological entities (e.g., miRNA and protein) and *E* denotes the set of undirected edges. DeepWalk employs a stream of short random walks to extract potential associations between miRNAs and proteins from the global network. The series that a random walk starts with every node *v*_*i*_ are marked as *W*_*v*_*i*__. Moreover, it is a stochastic process with random nodes $W_{v_{i}}^{1}$, $W_{v_{i}}^{2}$, …, $W_{v_{i}}^{k}$, where $W_{v_{i}}^{k+1}$ is a node chosen randomly from the neighbors of node *v*_*k*_. When getting the random walk sequence for each node, it needs to measure the probability of a specific sequence. More formally, given a sequence of nodes $W_{1}^{n}$ = (*w*_0_, *w*_1_, *w*_2_, …, *w*_*n*_), where *w*_*i*_ ∈*V*, DeepWalk maximizes the Pr(*w*_*n*_|*w*_0_, *w*_1_, *w*_2_, …, *w*_*n*−1_) over all nodes. The idea is to calculate the possibility of observing node *v*_*i*_ given all the previous nodes traversed heretofore in the random walk:

(2) $$\begin{array}{r} \mathrm{Pr}\left(v_{i}|\left(v_{1},\;v_{2},\;\ldots ,\;v_{i-1}\right)\right) \end{array}$$

We introduced a mapping function Φ:*v ϵ V* ↦ *R*^|*V*| × *d*^ to stand for the potential social representation associated with each miRNA and protein in the graph. The next step involves estimating the likelihood:

(3) $$\begin{array}{r} \mathrm{Pr}\left(v_{i}|\left(\Phi \left(v_{1}\right),\;\Phi \left(v_{2}\right),\;\ldots ,\;\Phi \left(v_{i-1}\right)\right)\right) \end{array}$$

However, as the walk length increases, it becomes too expensive to calculate this conditional probability. According to a recent publication (Mikolov et al., 2013), DeepWalk uses one node to predict the context, both the left and right neighbor nodes of the given node, instead of using the context to predict next node. In terms of node feature modeling, it yields the following optimization problem:

(4) $$\begin{array}{r} \text{minimize }-\log\mathrm{Pr}\left(\left\{v_{i-w},\;\ldots ,\;v_{i+w}\right\}\backslash v_{i}|\Phi \left(v_{i}\right)\right) \end{array}$$

To solve the optimization problem, we then employed SkipGram, a computational language model based on neural network that maximizes the co-occurrence likelihood over the nodes that appear among the context of node *v*_*i*_ in the random walk sequence, to approximate the conditional probability in Equation 4 based on an independence assumption, as follows:

(5) $$\begin{array}{r} \mathrm{Pr}\left(\left\{v_{i-w},\;\ldots ,\;v_{i+w}\right\}\backslash v_{i}|\Phi \left(v_{i}\right)\right)=\;\prod_{\begin{array}{c} j=i-w\\ j\neq i \end{array}}^{i+w}\mathrm{Pr}\left(v_{j}|\Phi \left(v_{i}\right)\right) \end{array}$$

For each of all the possible associations between biological entities in the random walk among the context of node *v*_*i*_, we mapped each node *v*_*j*_ to its recent representation vector $\Phi \left(v_{j}\right)\in \;R^{d}$ and maximized the posterior distribution probability of its neighbors in the walk. To speed up the computing time, we used the Hierarchical Softmax to approximate the probability distribution (Morin and Bengio, 2005; Mnih and Hinton, 2009):

(6) $$\begin{array}{r} \mathrm{Pr}\left(v_{j}|\Phi \left(v_{i}\right)\right)=\;\prod_{l}^{\left\lceil \log|V|\right\rceil }\mathrm{Pr}\left(b_{l}|\Phi \left(v_{i}\right)\right) \end{array}$$

By assigning the nodes to the leaves of a binary tree, we turned prediction of the potential association between miRNAs and proteins into maximizing the probability of a given path in the hierarchy. The path to node *v*_*j*_ is represented as a sequence of tree nodes (*b*_0_, *b*_1_, …, *b*_⌈log|*V*|⌉_). Moreover, Pr(*b*_*l*_|Φ(*v*_*i*_)) can be simulated by a binary classifier as follows:

(7) $$\begin{array}{r} \mathrm{Pr}\left(v_{j}|\Phi \left(v_{i}\right)\right)=\;1/\left(1+e^{-\Phi \left(v_{i}\right)\times \text{Ψ}\left(b_{l}\right)}\right) \end{array}$$

where $\text{Ψ}\left(b_{l}\right)\in \;R^{d}$ denotes the representation traversed to tree node *b*_*l*_'s parent.

After each node completes the random walk process γ times, a matrix Φ *ϵ R*^|*V*| × *d*^, which denotes the latent representations of the global network, is obtained. The result is that, in the matrix, each row represents a low-dimensional representation vector of a miRNA or a protein in the network. The source code and data of PmiRGO are freely available at http://denglab.org/PmiRGO/.

#### Training the SVM-Based Classifier

Due to the lack of manually curated GO annotations for miRNAs, it is dissatisfactory to build miRNA function predictors based on the miRNAs directly. Therefore, we built the training data sets with GO annotations of proteins downloaded from GOA database (version 201010) (Huntley et al., 2009). Proteins with lengths 50–100 aa were selected and clustered with a sequence similarity of 90 percent (Deng et al., 2018). Moreover, only one protein was chosen as a representation from each cluster. The representations without at least a non-IEA (not inferred from electronic annotation) GO term were filtered. As a result, 243,561 proteins with Gene Ontology annotations were collected.

For each GO term, we trained a classifier with samples of proteins. More specifically, we constructed a true annotation set for a GO term consisting of proteins, which had the GO annotation, and a false annotation set of proteins where these proteins did not have this GO function. As GO ontology is considered as a directed acyclic graph where each term is related to one or more other terms in the same domain or other domain (Deng and Chen, 2015; Zeng et al., 2018), the protein related to a GO term was also related to the ancestors of the term. Therefore, the false annotation data set was composed of proteins associated with other GO terms (excluding annotated terms and their child nodes). Due to the false annotation set containing more protein-GO pairs than the true annotation set, we randomly selected an equal number of negative and positive samples.

Here we employed support vector machines (SVMs) to build the binary classifier (Yong-Xin et al., 2011). SVM is widely used in bioinformatics research in the fields of miRNA target prediction, miRNA identification (Wei et al., 2014), RNA methylation prediction (Chen et al., 2017), and protein folding (Li et al., 2016), and others (Xiao et al., 2017; Dao et al., 2018; Feng et al., 2018; Pan et al., 2018; Yang et al., 2018; Zhu et al., 2019). We used the radial basis function kernel (RBF) as the kernel function, which achieved a better performance. *C* is the penalty coefficient of SVM, which can be considered as the weight to adjust the preference of two indexes (interval size, classification accuracy) in the optimization direction. The higher the value of *C*, the easier the classifier was to overfit. On the contrary, the lower the value of *C*, the easier the classifier was to underfit. To obtain an optimal *C* of the SVM and γ of the kernel, the performance for each *C* and γ was evaluated by carrying out a 10-fold cross-validation.

## Results

### Benchmarks

To accurately evaluate the performance of PmiRGO, we created an independent test based on the GOA database (Ashburner et al., 2000; The Gene Ontology Consortium, 2017). It consisted of a total of 337 mature miRNAs (named as miRNA2GO-337), each of which had at least one curated GO annotation (not inferred from electronic annotation, non-IEA). The independent test dataset appears in the Supplementary Table 1.

### Evaluation Measures

In PmiRGO, the classifier predicted several probable GO terms with corresponding scores ranging from 0 to 1 for a specific miRNA. The scores denoted the degree of confidence for those GO terms. The final predictions depended on the selected threshold *t*. All GO terms predicted for each miRNA with scores equal to or greater than *t* and their ancestors in GO linked by “is a” and “has a” relationships were collected to build the set of predicted GO terms denoted as *P*(*t*) for each threshold *t*. We used *T* to denote the set of experimentally validated GO terms. We evaluated the performance of the prediction according to three widely used statistic indexes: recall, precision, and F-measure. The definitions of recall and precision are as follows:

(8) $$\begin{array}{r} Pre_{i}\left(t\right)=\;\frac{\sum_{g\in G}I\left(g\;\in \;P_{i}\left(t\right)\;\wedge g\;\in \;T_{i}\right)}{\sum_{g\in G}I\left(g\;\in \;P_{i}\left(t\right)\right)} \end{array}$$

(9) $$\begin{array}{r} Rec_{i}\left(t\right)=\;\frac{\sum_{g\in G}I\left(g\;\in \;P_{i}\left(t\right)\;\wedge g\;\in \;T_{i}\right)}{\sum_{g\in G}I\left(g\;\in \;T_{i}\right)} \end{array}$$

where *g* denotes a specific GO term, and *G* denotes the set of all GO terms used in our work. The indicator function *I*(*x*) is stated as follows:

(10) $$I\left(x\right)={\{}_{0\;x=false}^{1\;x=true}$$

After all the miRNAs had been predicted, the average precision for each threshold *t* could be calculated on *m*(*t*) miRNAs, each of which had at least one predicted GO term with a score greater than the threshold *t*. In the same way, the average recall could be calculated from the whole benchmark set of *N* miRNAs. The average precision and recall are defined as follows:

(11) $$\begin{array}{r} \mathrm{Pre}\left(t\right)=\;\frac{1}{m\left(t\right)}\times \sum_{i=1}^{m\left(t\right)}Pre_{i}\left(t\right) \end{array}$$

(12) $$\begin{array}{r} Rec\left(t\right)=\;\frac{1}{N}\times \sum_{i=1}^{N}Rec_{i}\left(t\right) \end{array}$$

Generally speaking, precision and recall are inversely related. It is not feasible to evaluate the performance of models according to a single precision or recall. To deal with this problem, the maximum F-measure over all thresholds was introduced for the overall evaluation of different models (Zhang J. et al., 2018). It combined the two metrics (precision and recall) to provide a single-score. The maximum F-measure is defined as follows:

(13) $$\begin{array}{r} F_{max}=\max\left(\frac{2\times Pre\left(t\right)\times Rec\left(t\right)}{Pre\left(t\right)+Rec\left(t\right)}\right) \end{array}$$

### The Effects of Feature Dimensions

As described above, the latent representations of each node in the network act as its low-dimensional topological features. The number of dimensions might have a significant effect on the functional annotations of miRNAs. To assess the influence of the hyper-parameter on the prediction performance, we performed an independent test on the miRNA2GO-337 dataset across a wide range of values for the dimensions. For simplicity, we preset the other parameters, including the number of walks started from one node (n), the walk length (t), and the window size (w), in DeepWalk. The three parameters were selected by conducting experiments of different parameter values and choosing the combination with the best performance (*n* = 100, *t* = 80, *w* = 16).

**Figure 2:**
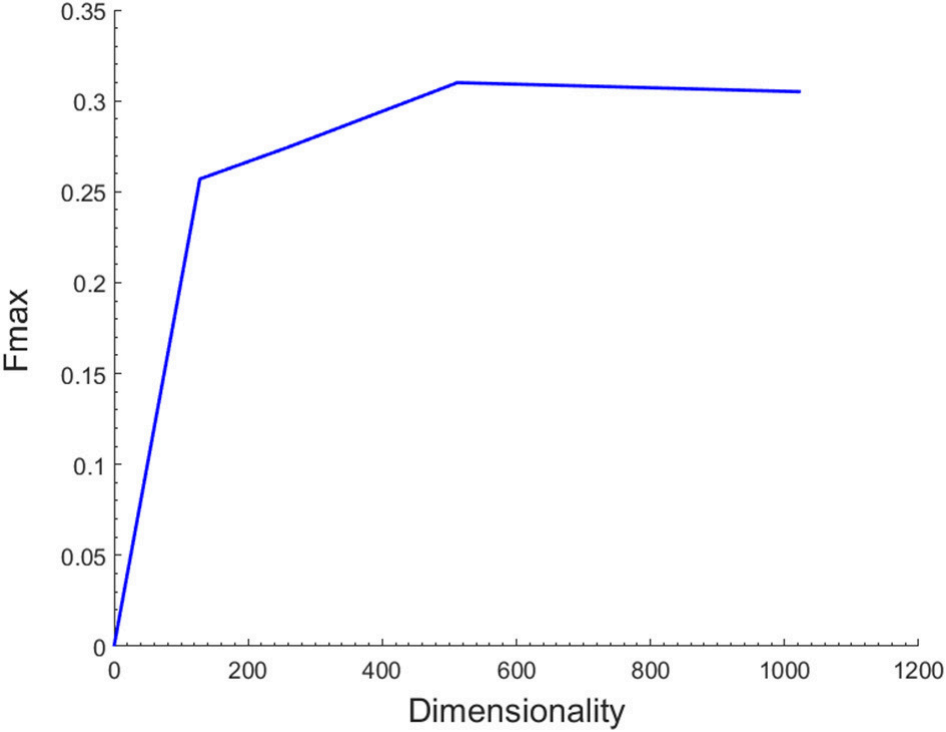
Effect of the number of different feature dimensions on function prediction. The maximum *F*-measure reaches its highest value 0.31 when the feature dimension is 512.

Figure 2 shows the *F*_*max*_ values when the number of dimensions ranges from 128 to 1024. The results demonstrated that the *F*_*max*_ reached the max value when the dimension increased to 512. However, as the dimension increased beyond this value, the performance decreased accordingly. Hence, 512 was chosen as the dimensions of the feature vector. It is important to note that the SkipGram model based on Hierarchical Softmax of DeepWalk algorithm is a neural network model and its output layer corresponds to a binary tree. Therefore, the dimensions of the latent representations of the model should be a power of two.

### The Effects of PPI Data

In our method, protein interaction data was incorporated to help improve the effectiveness of the functional annotations of the miRNAs. To confirm this, PmiRGO was carried out on two different network collocations: the global network (consisting of a miRNA co-expression network, miRNA-target interaction network, and PPI network), and the network without PPIs. The comparison was performed in terms of *F*_*max*_ when the parameters (*n, t, w, d*) were set to 100, 80, 16, and 512, respectively. The results are shown in Table 1. The *F*_*max*_ value was 0.31 for the global network and 0.252 for the network without PPIs. The performance increased ~23% with the addition of PPI data. This experiment demonstrated that integrating multiple types of information about other relevant biological entities (e.g., protein) resulted in a great improvement in the performance of predicting miRNA function.

**Table 1:** Performance evaluation of PPI network.

**Network**	**Precision**	**Recall**	***F_***max***_***
Without PPIN	0.328	0.205	0.252
Global network	0.351	0.277	0.310

### Comparison of Different Network Representation Algorithms

Recent studies have demonstrated that network representation learning is effective in machine learning, such as in tag recommendation (Tu et al., 2014), vertex classification (Sen et al., 2008), and link prediction (Lü and Zhou, 2011; Yang et al., 2015). Many methods have been proposed to address these issues, most of which investigate network structure for learning, such as DeepWalk (Perozzi et al., 2014), node2vec (Grover and Leskovec, 2016), hin2vec (Fu et al., 2017), and metapath2vec (Dong et al., 2017). DeepWalk used information extracted locally from the truncated random walks in order to learn potential representations. On the basis of DeepWalk, node2vec defined a strategy generating a sequence of bias random walk that used both BFS and DFS to retain different network structure information. Different from DeepWalk and node2vec, hin2vec, and metapath2vec have been proposed for heterogeneous information networks. They were designed to capture rich semantics by exploiting different types of relationships among nodes in forms of meta-paths.

**Figure 3:**
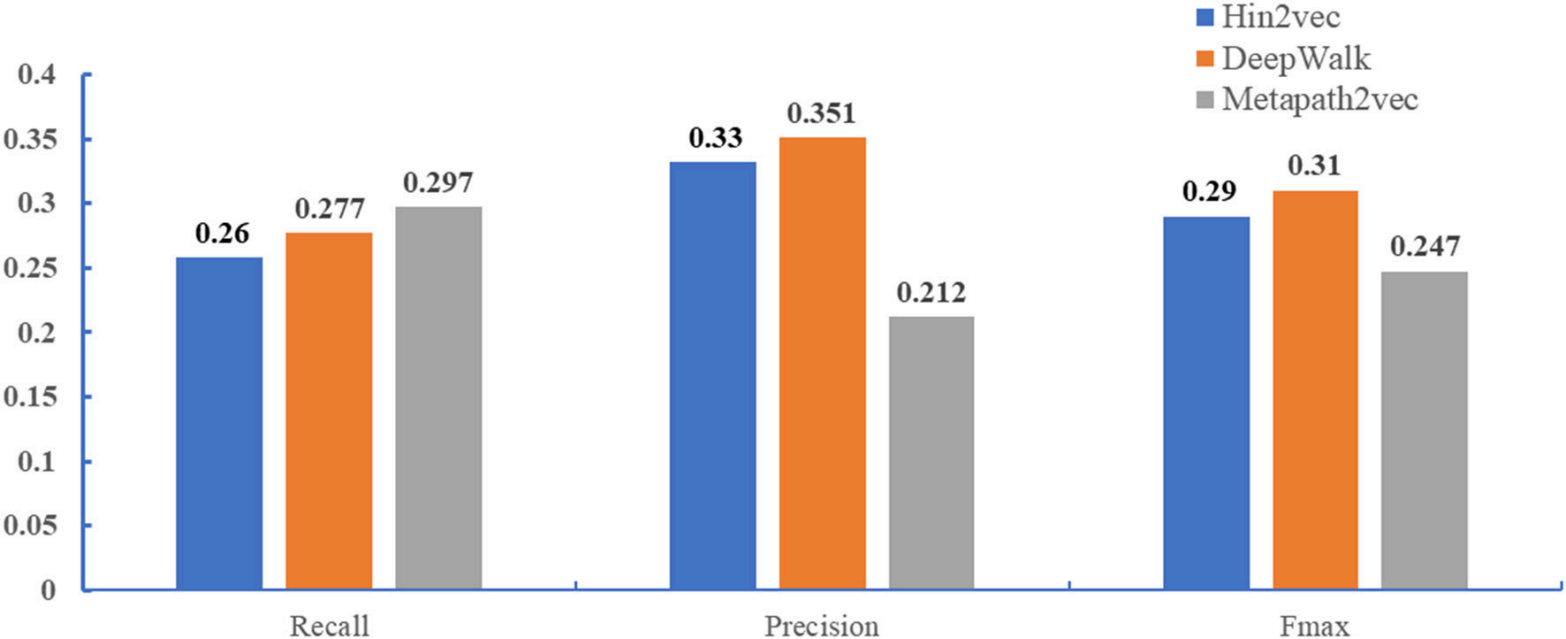
Performance comparison when different methods (DeepWalk, hin2vec, and metapath2vec) are employed to extract topological features. The performance of three methods were performed in the miRNA2GO independent dataset.

In this paper, we compared DeepWalk, hin2vec, and metapath2vec in terms of predicting GO annotations of miRNAs. For the sake of fairness, we used the same global network constructed above, multi-classification models, and benchmarks. Figure 3 demonstrates that DeepWalk significantly outperforms hin2vec and metapath2vec in terms of precision and *F*_*max*_. Hence, DeepWalk was employed to extract the topological features of our work.

### Performances

To evaluate the performance of PmiRGO further, we compared it with the state-of-the-art method miEAA (Backes et al., 2016). MiEAA is a tool that uses enrichment analysis to perform the functional analysis of sets of miRNAs based on GeneTrail (Backes et al., 2007). Compared to GeneTrail, miEAA was designed for human miRNA precursors and mature miRNAs. The miRNA2GO-337 dataset was utilized to assess the performance of different methods. Since 53.5% of the functional annotations of miRNAs are biological process (BP) terms, according to the statistics of Gene Ontology Consortium database (Ashburner et al., 2000), and since miRNAs are involved in the biological process when they have interactions with other entities, we only evaluated the performance in terms of BPs.

**Figure 4:**
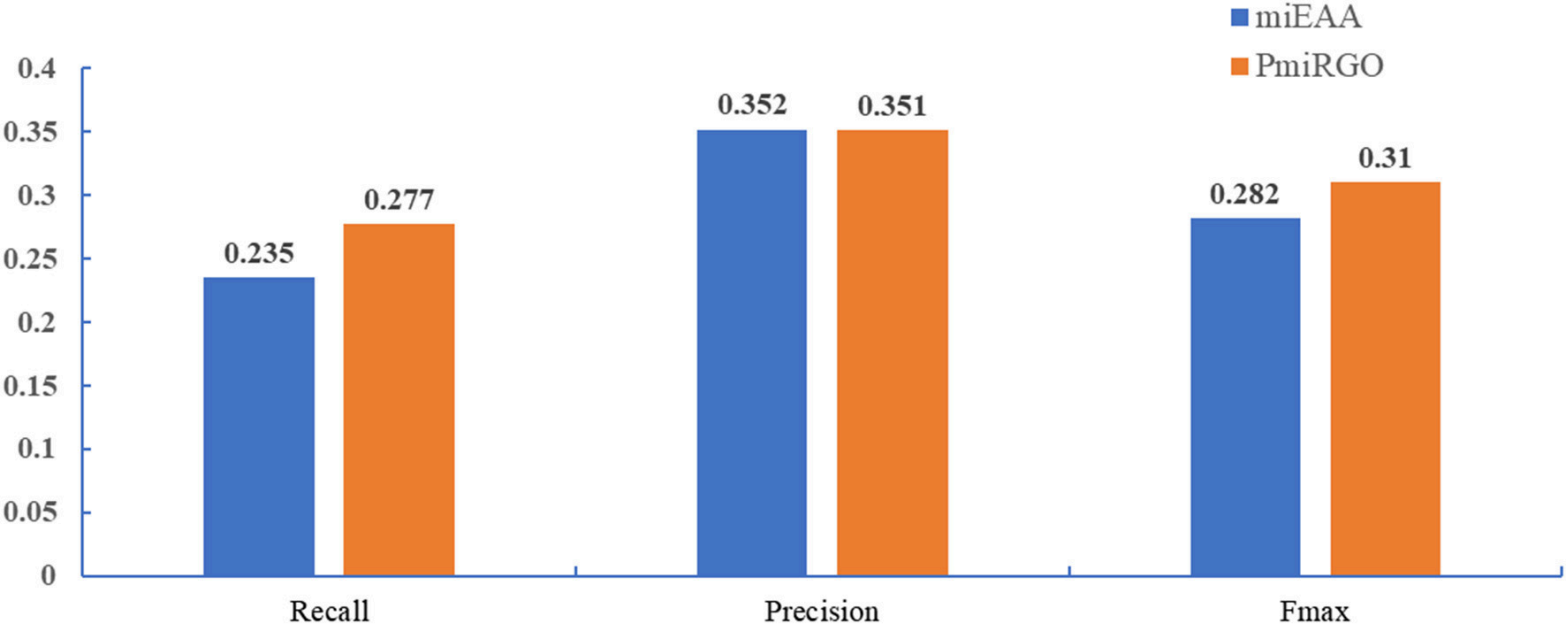
Performance comparison between PmiRGO and miEAA in terms of recall, precision, and *F*_max_.

**Figure 5:**
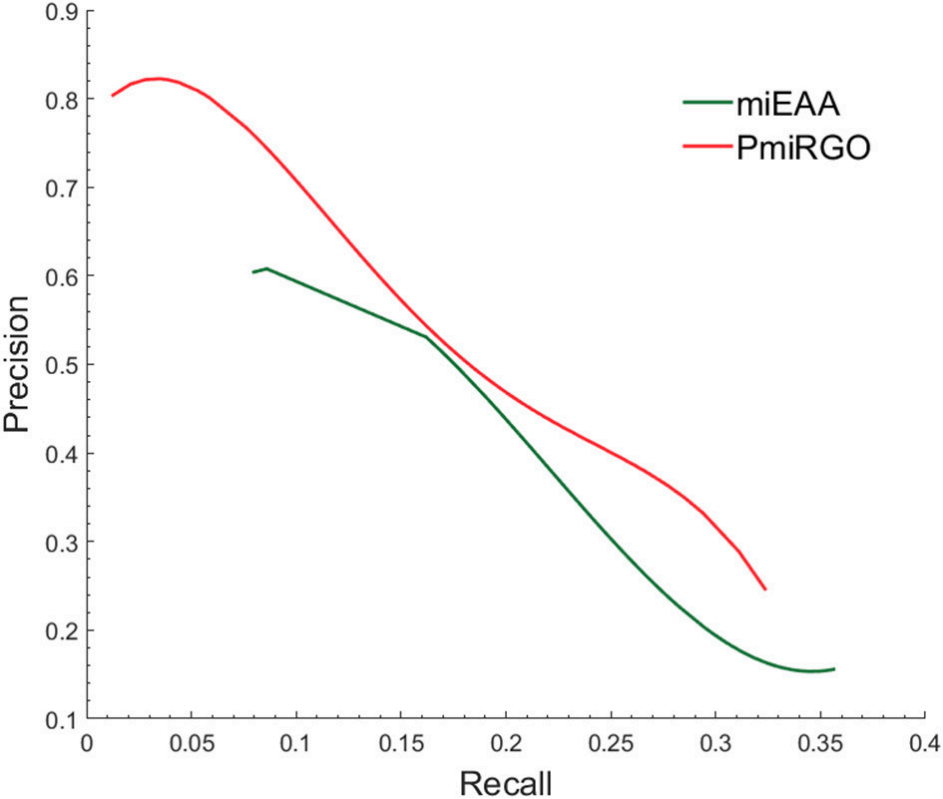
Precision-recall curves of PmiRGO and miEAA. The figure shows the performance comparison of PmiRGO with miEAA on the miRNA2GO-337 dataset for BP terms.

The prediction performance of the two methods is presented in Figure 4. It is quite apparent that PmiRGO outperforms miEAA. For the metric *F*_*max*_, PmiRGO achieved 0.310 *F*_*max*_ on BP terms and had an increase of 0.03 *F*_*max*_, while miEAA reached 0.282 *F*_*max*_. Also, the recall of PmiRGO reached 0.277 when the *F*_*max*_ achieved the highest value, and the recall of miEAA was 0.235. Figure 5 shows that the precision-recall curve of PmiRGO is entirely above the curve of miEAA, which means that our method significantly outperforms miEAA. We calculated the *P*-value with two-tailed, paired *t*-test to compare the performances of our PmiRGO method and MiEAA. For each time, we randomly selected 50 miRNAs from the miRNA2GO-337 dataset and calculated the *F*_*max*_ scores for both PmiRGO and MiEAA. We repeated the procedure for 30 times and obtained 30 paired *F*_*max*_ scores. We calculated the *P*-value using MATLAB. A *P*-value score of 0.05 was used to denote statistical significance. The *F*_*max*_ of our PmiRGO method was higher than that of MiEAA, a difference that was statistically significant (*P* = 1.86e-05).

**Figure 6:**
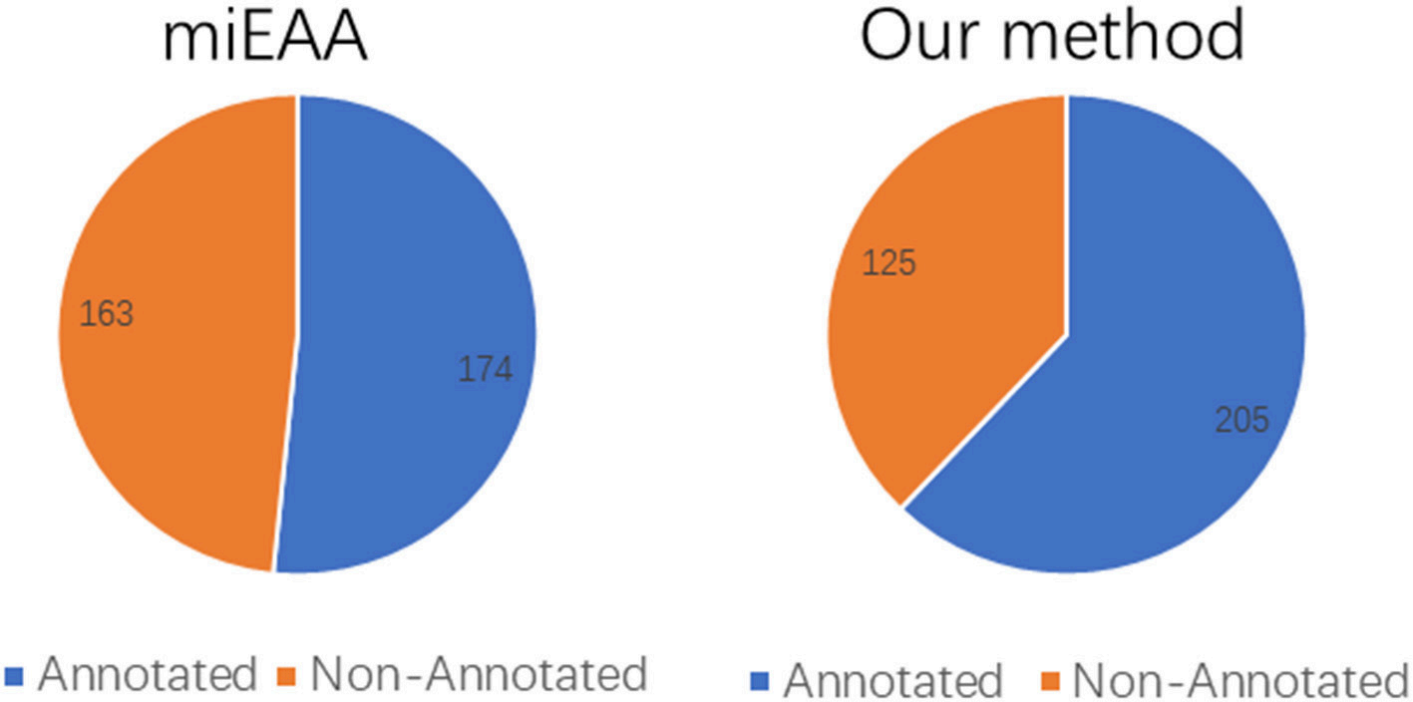
Performance comparison of coverage, on the independent dataset miRNA2GO-337. Among these 337 human miRNAs, 205 miRNAs were annotated by PmiRGO, while only 174 miRNAs were annotated by miEAA.

Moreover, the coverage of the two prediction methods on the miRNA2GO-337 dataset was compared. The coverage is defined as the number of miRNAs predicted correctly, a measure that reflects robustness. As presented in Figure 6, PmiRGO correctly annotated 205 miRNAs out of 337 miRNA samples, while miEAA successfully predicted 174 miRNAs, demonstrating that our method is more robust than miEAA.

### Case Study

To illustrate the performance of this prediction method in a real case study, we applied PmiRGO to predict the functions of miRNA has-miR-124-3p. miRNA has-miR-124-3p plays an essential role in mediating tumor growth and the occurrence and development of cancer with high genetic conservation. Recent studies have used high-throughput sequencing to demonstrate that hsa-miR-124-3p has differential expression in normal brain tissue and glioblastoma multiforme (GBM). Moreover, has-miR-124-3p overexpression expressively inhibits GBM cell proliferation, migration, and tumor angiogenesis, which results in cell cycle arrest and GBM apoptosis putatively via the activation of the NRP-1-mediated PI3K/Akt/NFκB pathway in GBM cells, as well as suppressing tumor growth and reducing tumor angiogenesis (Zhang G. et al., 2018). Moreover, hsa-miR-124-3p regulates the expression of the CD151 protein by inosculation with the 5′UTR to take part in the development of gastric cancer (Sheng et al., 2009).

As a result, has-miR-124-3p annotated 250 GO terms in total, the top 31 of which had a probability score >0.9, as shown in Table 2. Of the four most probable GO Terms, GO:0006915 (apoptotic process), responsible for the process of programmed cell death when a cell receives an internal or external signal, and GO:0006725 (cellular aromatic compound metabolic process), the chemical reactions and pathways involving aromatic compounds, were indirectly related with the occurrence and development of diseases, particularly cancer and tumors. In addition, the predicted GO Terms GO:0008219 (cell death) (ranked 5th), GO:0048468 (cell development) (ranked 7th), and GO:0009987 (cellular process) (ranked 30th) were associated with adenocarcinoma of the lung, breast neoplasms, and colonic neoplasms. Moreover, those GO terms related to metabolic processes, such as GO:0006259 (DNA metabolic process) (ranked 9th), GO:0019216 (regulation of lipid metabolic process) (ranked 12th), and GO:0031323 (regulation of cellular metabolic process) (ranked 15th), were associated with the production of the gene products TCEAL7 and TNFRSF1A, which may promote the occurrence of prostatic neoplasms, lung diseases, and gastric cancer.

**Table 2:** The top 31 GO terms predicted for miRNA has-miR-124-3p.

**Rank**	**GO term**	**GO name**
1	GO:0006915	Apoptotic process
2	GO:0006725	Cellular aromatic compound metabolic process
3	GO:0003677	DNA binding
4	GO:0051234	Establishment of localization
5	GO:0008219	Cell death
6	GO:0048856	Anatomical structure development
7	GO:0048468	Cell development
8	GO:0043169	Cation binding
9	GO:0006259	DNA metabolic process
10	GO:0007165	Signal transduction
11	GO:0045664	Regulation of neuron differentiation
12	GO:0019216	Regulation of lipid metabolic process
13	GO:0006810	Transport
14	GO:0008104	Protein localization
15	GO:0031323	Regulation of cellular metabolic process
16	GO:0009892	Negative regulation of metabolic process
17	GO:0005102	Signaling receptor binding
18	GO:0042176	Regulation of protein catabolic process
19	GO:0050769	Positive regulation of neurogenesis
20	GO:0006508	Proteolysis
21	GO:0016477	Cell migration
22	GO:0008202	Steroid metabolic process
23	GO:0008168	Methyltransferase activity
24	GO:0051252	Regulation of RNA metabolic process
25	GO:0009411	Response to UV
26	GO:0014902	Myotube differentiation
27	GO:0045596	Negative regulation of cell differentiation
28	GO:0005515	Protein binding
29	GO:0055085	Transmembrane transport
30	GO:0009987	Cellular process
31	GO:0007224	Smoothened signaling pathway

## Discussion

Computational function prediction of miRNAs by integrating varieties of miRNA-related biological information is emerging as a tool to elucidate the role of miRNAs in development and for inferring the biological functions of miRNAs. In our work, we proposed a novel approach, PmiRGO, to predict their function. Specifically, we constructed a global heterogeneous network by integrating expression profiles, miRNA-target interactions, and PPI data. Then, DeepWalk, an approach used for learning online social representations, was employed to learn the latent network features of the global network. Finally, we employed SVM to build multi-classification models for predicting the GO annotations.

In terms of the performance, PmiRGO was used to evaluate the independent dataset miRNA2GO-337. In terms of *F*_*max*_ and coverage, PmiRGO outperformed miEAA. Moreover, the results demonstrate that the protein interaction data contributes to the improvement of prediction performance for miRNAs. The great performance of our method can be attributed to several factors. At first, the experimentally validated miRNA-target gene interactions, manually curated from reporter assay, blot, and microarray experiments were utilized. More reliable and positive information significantly improves the performance of PmiRGO. Then, we used the miRNA expression profiles to construct a miRNA co-expression network, which is useful for predicting the miRNAs involved in co-regulating one target gene. Finally, the PPI network was introduced to the global network, allowing the performance of function prediction to benefit from the variety of biological entities.

However, there are still further improvements to be made to our method. Firstly, the experimentally validated miRNA-target gene interactions were sparse. A greater number of validated interactions could enhance the effect of PmiRGO further. Secondly, the expression profiles we used covered only a part of human miRNAs, and the coverage of the expression information was not enough. As such, more reliable miRNA expression profiles need to be collected. Thirdly, more types of biological entities could also be introduced to the global network. Others works, including miRNA family information (Zou et al., 2014) and miRNA-disease networks (Zou et al., 2016; Liao et al., 2018; Zeng X. et al., 2018), would also be useful in this study. This should be the focus of future works.

## Author Contributions

LD, JW, and JZ conceived this work and designed the experiments. LD and JW carried out the experiments. LD, JW, and JZ collected the data and analyzed the results. LD, JW, and JZ wrote, revised, and approved the manuscript.

### Conflict of Interest Statement

The authors declare that the research was conducted in the absence of any commercial or financial relationships that could be construed as a potential conflict of interest.

## Supplementary Material

Supplementary Table 1 The miRNA2GO-337 dataset.
